# Turkish Baths

**Published:** 1872-05

**Authors:** 


### TURKISH BATHS

May be very good in some forms of disease, when they are judiciously administered; but they so frequently kill people that it would be better never to take one, unless by the special advice of your family physician, and even then, it should be submitted to only when under the special, personal superintendence of a respectable, educated, medical man.

Miss Lillie Peckham, a young lady of great promise, and of unusual talents and ability, took a Turkish Bath at Milwaukee, and was so debilitated that she died in a few days. Dr. Trall, one of the most able hydropathists, says that more than twenty seriously injurious or fatal cases have come to his personal knowledge, not from the dangerous nature of the baths themselves, but from their injudicious administration by the hands of ignorant hirelings.

Turkish baths are very good for dirty people, such as have not had a good cleaning off in a year; but we never could imagine the utility of putting a decent man into a steam-boiler hot enough to skin a lobster, and then filing off all his hide, to the very quick, by kneadings and remorseless scrubbings.

